# Cognitive ability following exposure to parental mental disorders and other childhood adversities: a population-based cohort study of Danish males in late adolescence

**DOI:** 10.1007/s00431-026-07102-2

**Published:** 2026-05-28

**Authors:** Tanja Gram Petersen, Gunhild Tidemann Okholm, Kirstine Davidsen, Rikke Wesselhoeft, Merete Osler, Trine Munk-Olsen, Mette Bliddal

**Affiliations:** 1 Research Unit OPEN, Department of Clinical Research, University of Southern Denmark, Odense, Denmark; 2 Center for Clinical Research and Prevention, Bispebjerg and Frederiksberg Hospitals, The Capital Region, Frederiksberg, Denmark; 3 The National Institute of Public Health, University of Southern Denmark, Copenhagen, Denmark; 4 Department of Child and Adolescent Psychiatry, Mental Health Servicesin the, Region of Southern Denmark , Odense, Denmark; 5 Department of Psychology, University of Southern Denmark, Odense, Denmark; 6 Research Unit of Child and Adolescent Psychiatry, Department of Clinical Research, University of Southern Denmark, Odense, Denmark; 7 Clinical Pharmacology, Pharmacy and Environmental Medicine, Department of Public Health, University of Southern Denmark, Odense, Denmark; 8 Section of Epidemiology, Department of Public Health, University of Copenhagen, Copenhagen, Denmark; 9 Department of Clinical Research, University of Southern Denmark, Odense, Denmark; 10 Department of Gynecology and Obstetrics, Odense University Hospital, Odense, Denmark

**Keywords:** Cohort study, Cognitive ability, Childhood adversities, Mental disorders

## Abstract

The purpose of the study is to examine the association between parental mental disorders diagnosed before age six and cognitive ability at age 18, using data from a population-based cohort of male adolescents attending military conscription. Further, we tested whether other childhood adversities modified this association. All males born in Denmark 1996–2001 were linked to national registers. Parental mental disorders were identified from psychiatric hospital diagnoses. We assessed other childhood adversities before age six, including family health-related, socioeconomic, and family instability, as well as the number of adversities experienced. Cognitive scores from the mandatory military conscription were standardized (mean 100, SD = 15). Associations were estimated using multiple linear regression, and effect modification was evaluated using Wald tests. We included 125,791 males (mean age 18 years, SD = 0.66), of whom 6708 had a parent with a mental disorder. Parental mental disorders were associated with a 0.91-point reduced cognitive ability score (95% confidence interval − 1.3, − 0.53). This association was not observed in adolescents who lived with only one parent (− 0.35 (− 0.85, 0.15)), those placed in out-of-home care (3.2 (0.59, 5.8)), and those with ≥ 3 additional adversities before age six. Effect modification tests were statistically significant for these groups (*p* < 0.01).

*Conclusion*: Parental mental disorders before age six were associated with a modest reduction in cognitive ability in males at age 18, compared with those without parental mental disorders. Our findings show the association varies across family contexts, although unmeasured genetic and environmental confounding should be considered in interpreting these results.

**Table:** 

**What is Known:** • *Parental mental disorders are common and linked to lower cognitive ability in offspring*. • *Childhood adversities often cluster within families and are associated with poorer cognitive outcomes*.	
**What is New:** • *In this nationwide Danish cohort, parental mental disorders before age six were associated with slightly lower cognitive ability at age 18*. • *This association was not observed among adolescents with > 2 additional adversities, those who lived with only one parent, or those placed in out-of-home care before age six, suggesting the association varies across family context*.

**Supplementary Information:**

The online version contains supplementary material available at 10.1007/s00431-026-07102-2.

## Introduction

Parental mental disorders are common and represent a significant source of adversity in childhood with potential long-term consequences for the offspring’s development [1, 2]. In Denmark and Sweden, more than one in ten children grow up with a parent affected by a mental disorder requiring hospital care [3, 4]. Parental mental disorders have been associated with reduced cognitive ability in offspring in early childhood, although findings are inconsistent [5–8]. In addition, parental mental disorders frequently coexist with other stressful conditions, such as family health problems, socioeconomic disadvantage, and family instability, reflecting the tendency of childhood adversities to cluster within families [1, 2, 9–12].

Different childhood adversities have independently been linked to adverse health and cognitive outcomes in offspring, with poorer outcomes observed among children experiencing multiple adversities [1, 2, 9, 13–16]. The potential modifying role of other childhood adversities in the association between parental mental disorders and offspring cognitive ability has largely been overlooked in previous studies [5–8, 17, 18]. One possibility is that exposure to parental mental disorders in combination with additional adversities leads to a cumulative burden of disadvantage, whereby early and co-occurring adversities compound over time and result in poorer cognitive development, consistent with cumulative disadvantage theory [19].

Using a population-based cohort of Danish male adolescents, this study aimed to examine the association between parental mental disorders diagnosed in hospital settings before the offspring’s age six and cognitive ability at age 18. Further, we aimed to study whether any observed association was modified by other childhood adversities, including family health-related, socioeconomic, and family instability factors. We focused on early childhood adversities and described patterns of association without inferring causality.

## Methods

We conducted a population-based cohort study of Danish male adolescents who completed the Danish military cognitive ability test between 2014 and 2019. We used the Danish nationwide registry system, in which all residents are assigned a unique personal identification number to link individual-level data across registers [20, 21]. Individuals were linked to their legal parents and siblings through the Danish Civil Registration System [20, 21].

### Study population

**Fig. 1:**
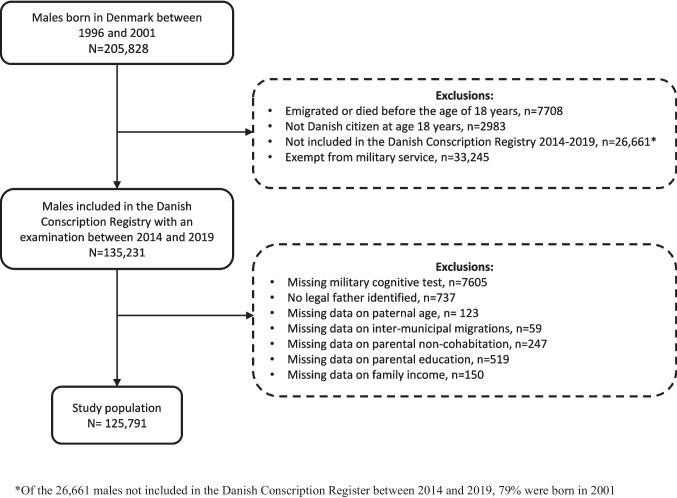
Flowchart of the study population

We initially identified 205,828 males born in Denmark between 1996 and 2001 using the Danish Medical Birth Register (Fig. 1) [22]. We excluded individuals who emigrated or died before the age of 18 years (*n* = 7708) and individuals who were not Danish citizens (*n* = 2983). We also excluded individuals not contained in the Danish Conscription Registry (31) between 2014 and 2019 (*n* = 26,661, of whom 79% were born in 2001), which keeps information on the examinations for military service eligibility. In Denmark, all 18-year-old males with Danish citizenship are required by law to attend this examination, while females born before July 2007 may participate on a voluntary basis. Examination can be postponed until age 26 at the latest. Thus, the Danish Conscription Registry includes nearly all eligible males, except those postponing examination. We also excluded those who were exempt from military service and had not undergone an examination for military eligibility (*n* = 33,245) due to documented medical conditions such as epilepsy, type 1 diabetes, intellectual disabilities, severe mental disorders, or other similar diagnoses (31). Further exclusions were made for individuals with missing data on the military cognitive ability test (*n* = 7605), lacked a legally registered father (*n* = 737), or had missing data on any covariate or childhood adversity (*n* = 1098). Missing data on covariates and childhood adversities were minimal (< 1%) and handled using complete case analysis. The missingness was likely related to recorded characteristics in the registers, and any resulting bias is expected to be negligible. Missing outcome data primarily reflected exemption from conscription due to documented medical conditions and were therefore unlikely to be missing at random.

### Parental mental disorders

The primary childhood adversity was parental mental disorders within the first 6 years of life (corresponding to the typical pre-school age in Denmark). We defined parental mental disorders as any in- or outpatient psychiatric hospital diagnosis of International Classification of Diseases version 10 (ICD-10) codes: F1–6*, F8* recorded for any of the parents in the Danish National Patient Registry [23]. Parental mental disorder was assessed as a dichotomous variable (present/absent) based on diagnoses given from the date of the child’s birth to their sixth birthday.

### Other childhood adversities

Other childhood adversities within the first 6 years of life were defined within the following three groups: family health-related adversities, socioeconomic disadvantage, and family instability. The adversities selected were chosen on the basis of their association with long-term offspring outcomes [1, 2, 24]. Table 1 provides an overview of the definitions of the selected childhood adversities and the corresponding data sources. Besides assessing each adversity type individually, we also evaluated the number of additional adversities (0, 1, 2, 3, ≥ 4).

**Table 1:** Childhood adversities beyond parental mental disorders—definitions and data sources

Childhood adversities	Definition (categorization)^a^	Data source
**Health-related adversities**		
Death of a parent	Recorded death date of a parent (no/yes)	The Danish Civil Registration System [20]
Death of a sibling	Recorded death date of a sibling (no/yes)	The Danish Civil Registration System
Parental somatic illness	A parent diagnosed with at least one ICD-10 code in the Charlson Comorbidity Index [50] (no/yes)	The National Patient Registry [23]
Sibling somatic illness	A sibling diagnosed with one of the following ICD-10 codes: C00–C96 (malignant neoplasm), Q20–Q28 (congenital anomalies of the heart and circulatory system), Q00–Q07 (congenital anomalies of the nervous system), G80–G83 (cerebral palsy), G40–G41 (epilepsy), I42–I43 (cardiomyopathy), E75 (congenital disorders of lipid metabolism) [24] (no/yes)	The National Patient Registry
Sibling mental disorders	A sibling diagnosed with one of the following ICD-10 codes: F1–9* (no/yes)	The National Patient Registry
**Socioeconomic disadvantage**		
Poverty	Family income below the 33% percentile of the national family income for three consecutive years (no/yes)	The Income Statistics Register [32]
Parental long-term unemployment^b^	A parent experiencing unemployment for at least 12 months for two consecutive years (no/yes)	The Danish Register for Evaluation of Marginalization (DREAM) database
**Family instability**		
Parental non-cohabitation	At least one parent residing at a different address (no/yes)	The Danish Civil Registration System
Inter-municipal migrations	Number of changes in the registered municipality (0–2/≥ 3)	The Danish Civil Registration System
Children placed outside of own home	Registered placement outside the home (no/yes)	The Children and Youth in Out-of-Home Care Register

^a^All adversities were measured from birth until age 6 years

^b^Data on long-term unemployment was only available from 1998 and onwards

### Outcome: offspring cognitive ability test scores

The outcome was cognitive ability test scores at age 18 years, measured using the Danish military cognitive ability test, Børge Priens Prøven (BPP), obtained from the Danish Conscription Registry [25]. The test is administered as part of the Danish Conscription Board Examination and comprises four subtests designed to assess logical, verbal, numerical, and spatial reasoning abilities [26, 27]. The internal reliability of the four subtests has been shown to be high (Cronbach’s alpha ranging from 0.7 to 0.8) [26]. The total BPP score ranges from 0 to 78 [25] and demonstrates strong correlations (0.82) with the full-scale Wechsler Adult Intelligence Scale [28] as well as substantial test–retest correlations of around 0.80 over a 40-year period [29, 30]. We standardized the BPP scores to a sample mean of 100 and an SD of 15 to aid comparison with studies reporting intelligence quotient points. The distribution was assessed visually using a histogram, which indicated no substantial deviations from normality.

### Baseline characteristics

Information on offspring birth year (1996–2001) and whether they were firstborn (“yes,” “no”) was obtained from the Danish Medical Birth Register [22]. Additional characteristics were retrieved from the Danish Civil Registration System [20] and the Danish Education Register [31] at the time the child was born and from the Income Statistics Register [32] for the year preceding birth. These included maternal and paternal age (modeled using restricted cubic splines); parental citizenship (“Danish,” “other Western” if at least one parent was Western and none was non-Western, “non-Western” if at least one parent was non-Western); parental highest attained educational level (“lower secondary,” “upper secondary,” and “post-secondary”), based on the parent with the highest level of education; and family equivalent disposable income, categorized into national tertiles (“lowest,” “middle,” and “highest”).

### Statistical methods

Descriptive characteristics of the study population by parental mental disorders were reported as frequencies and percentages for categorical variables and as medians with interquartile ranges for continuous variables. Associations were assessed using linear regression models, treating cognitive ability test scores as a continuous outcome. Results are presented as mean differences in cognitive ability test scores, with 95% confidence intervals (CIs) calculated using robust variance estimators to account for clustering among siblings.

In our primary analyses, we estimated the association between parental mental disorders and cognitive ability test scores overall and separately for adolescents who had experienced each additional adversity and those who had not [33]. Effect modification was formally tested by including interaction terms between parental mental disorders and each adversity, with statistical significance evaluated using Wald tests (*p* < 0.05). We further examined how the number of additional adversities modified the association, by including the interaction-term between parental mental disorder and the count of additional adversities in a statistical model. Covariates in the adjusted model for the overall association between parental mental disorders and offspring cognitive test scores were birth year, firstborn status, parental highest educational level, maternal and paternal age, family income, parental citizenship, sibling death, parental somatic illness, sibling somatic illness, sibling psychiatric illness, and parental non-cohabitation. Directed acyclic graphs guided the selection of potential confounders, and Appendix S1 details the covariates included in models assessing associations within each stratum of childhood adversities.

Several additional analyses were conducted to assess robustness and examine specific subgroups.

First, we examined the associations of family health-related adversities, socioeconomic disadvantage, family instability, and the number of adversities with cognitive ability test scores—both overall and stratified by parental mental disorders.

Second, we examined the association between parental mental disorders and offspring cognitive test scores across categories reflecting the child’s living situation in relation to the parent with a mental disorder (living apart from the affected parent or the affected parent having died).

Third, we extended the exposure window to encompass adversities experienced within the first 12 years of life, rather than just the first 6 years of life. Age 12 years was chosen as it marks the transition from early to later primary school in Denmark and the onset of adolescence.

Finally, to improve comparability with studies focusing on specific diagnostic groups, we conducted separate analyses for anxiety and depressive disorders (ICD-10 codes F32–F33*, F40–F48*), bipolar disorders (ICD-10 codes F30–F31*), and schizophrenia (ICD-10 codes F20–F29*).

## Results

### Characteristics of the study population

The study included 125,791 male adolescents. Of these, 6708 (5.3%) had at least one parent diagnosed with a mental disorder before their sixth birthday. A slightly higher proportion (5.7%) of individuals born between 1999 and 2001 had a parent diagnosed compared to those born between 1996 and 1998 (5.1%) (Table 2). Being firstborn, parental non-Western citizenship, low parental educational level, low family income, and younger maternal age were associated with a higher prevalence of parental mental disorders.

**Table 2:** Characteristics at birth according to parental mental disorders before age six

Characteristics	Parental mental disorders	
	No *N* = 119,083	Yes *N* = 6708
	*n* (%)	*n* (%)
Birth year		
1996–1998^a^	71,561 (95)	3834 (5.1)
1999–2001^a^	47,522 (94)	2874 (5.7)
Firstborn	50,354 (42)	2955 (44)
Parental citizenship		
Danish	106,255 (89)	5493 (82)
Other Western	3933 (3.3)	288 (4.3)
Non-Western	8895 (7.5)	927 (14)
Parental highest obtained education		
Lower secondary	11,340 (9.5)	1516 (23)
Upper secondary	58,616 (49)	3366 (50)
Post-secondary	49,127 (41)	1826 (27)
Family income tertiles		
Lowest tertile	21,365 (18)	2416 (36)
Middle tertile	38,080 (32)	2357 (35)
Highest tertile	59,638 (50)	1935 (29)
Maternal age, median [Q1; Q3]	29 (26; 32)	28 (24; 32)
Parental age, median [Q1; Q3]	31 (28; 35)	31 (27; 35)

^a^Row percentage

### Parental mental disorders and cognitive ability test scores by other childhood adversities

**Fig. 2:**
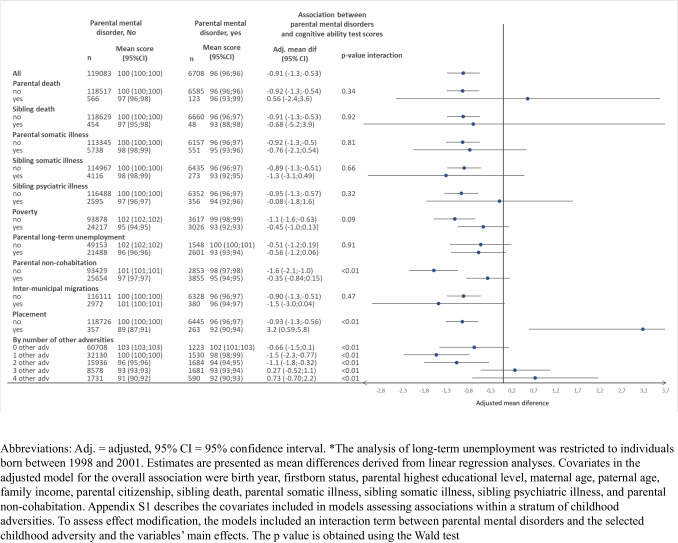
Associations between parental mental disorders in the first 6 years of life and cognitive ability test scores in males at age 18 years, overall and stratified by other childhood adversities

The mean age for the Danish Conscription Board Examination was 18 years (SD = 0.66). Figure 2 shows the adjusted mean differences in cognitive ability test scores associated with parental mental disorders, both overall and within groups defined by other family health-related adversities, socioeconomic disadvantages, family instability, and the number of other adversities. After adjustment, adolescents whose parents had mental disorders had on average 0.91 points lower cognitive ability test scores at age 18 compared with those whose parents had no recorded mental disorders (95% CI − 1.3, − 0.53). Table S1 also shows the crude estimates.

After stratification by individual adversities before age six, parental mental disorders were associated with reduced cognitive ability test scores in adolescents among those without the additional adversity, whereas estimates were less precise among those with the adversity (Fig. 2). Tests indicated statistically significant effect modification (*p* < 0.01) for analyses stratified by parental non-cohabitation and placement in out-of-home care. Here, parental mental disorders were associated with reduced cognitive ability test scores among adolescents who lived with both parents up to age six (mean difference − 1.6 (95% CI − 2.2, − 1.0)) but not among those living only with one parent (mean difference − 0.35 (95% CI − 0.84, 0.15)). Among adolescents living at home until age six, parental mental disorders were associated with reduced scores (mean difference − 0.93 (95% CI − 1.3, − 0.56)), whereas the association reversed among those placed in out-of-home care (mean difference 3.2 (95% CI 0.59, 5.8)). Associations also differed by the number of additional adversities (test for effect modification, *p* < 0.01): Associations were observed among adolescents with up to two childhood adversities but not among those with three or more (Fig. 2).

### Additional analyses

All single childhood adversities were individually associated with reduced cognitive ability test scores, except intermunicipal migrations; adolescents who had moved three or more times before age 6 years had higher scores than those who moved fewer times (Table S2). Analyses confirmed that this finding was not influenced by a greater prevalence of migration among higher socioeconomic groups (results not shown). Moreover, when examining the number of additional childhood adversities as the exposure, the mean cognitive ability test score was lower as the number of additional childhood adversities increased, with the strongest associations observed in the group of adolescents whose parents did not have mental disorders (Table S3).

The association between parental mental disorders and cognitive ability test scores did not differ according to whether the adolescent lived with the affected parent or based on whether the affected parent had died before age six (Table S4). Moreover, when we extended the exposure window for mental disorders and childhood adversities to 12 years, the associations between parental mental disorders and cognitive ability test scores remained consistent with the primary findings. However, we did not observe variation in the association in strata defined by different numbers of additional adversities as in the primary analyses (Tables S5). Parental anxiety/depression and bipolar disorders were generally not associated with reduced cognitive ability test scores (Table S6-7), while schizophrenia showed associations more consistent with the primary analyses (Table S8).

## Discussion

This register-based Danish cohort study found a modest association between parental mental disorders diagnosed at hospital settings before age six and reduced cognitive ability at age 18 among male adolescents. We found no association among offspring living with only one parent and among those experiencing three or more additional adversities. In adolescents placed in out-of-home care before age six, parental mental disorders were associated with higher cognitive scores, although estimates were imprecise.

Children’s social and family environments impact cognitive development, and parental mental disorders can be a major source of stress for the child with potential long-term effects on offspring cognition [9, 17, 34–37]. Our findings are consistent with studies showing an association between parental mental disorders and lower cognitive ability [5–7]. However, both psychiatric disorders and cognitive ability have substantial heritable components, and shared genetic and family environmental factors may partly explain the observed association [38–42]. The absence of an association between parental mental disorder and cognitive ability in certain family contexts in our study could reflect environmental contribution to the association. Nonetheless, the impact of unobserved factors, including genetic influences, cannot be dismissed.

Childhood adversities often co-occur [1], but it remains unclear how additional adversities modify the association between parental mental disorders and cognition. Consistent with our findings, socioeconomic factors did not modify the association between maternal depressive symptoms and cognition in children aged 5 and 8 years in two previous studies [17, 18]. We showed parental mental disorders were associated with higher cognitive ability among adolescents placed in out-of-home care before age six. However, the sample size was small leading to wide confidence intervals. Children placed in out-of-home care represent a selected group with complex and often severe childhood adversities. The observed association may therefore be affected by limited statistical accuracy, selection mechanisms, but also confounding factors, including genetic influences, and should be interpreted with caution.

Previous studies show that exposure to multiple adversities is generally associated with poorer cognitive outcomes [1, 13, 16, 43]. We also found that children with most adversities had the lowest cognitive ability scores, but the association between parental mental disorders and cognitive ability was absent among adolescents experiencing three or more additional adversities before age six. One possible explanation is that parental mental disorders and other adversities may reflect overlapping heritable traits, as well as overlapping stress-related and environmental mechanisms. As a result, additional adversities may not further reduce cognitive ability beyond what is already associated with parental mental disorders [38–40, 44, 45].

Early childhood is characterized by the child’s strong reliance on the parents and rapid brain development [9, 46]. We therefore expected that including adversities up to age 12 years, rather than only up to six years as in the primary analyses, would dilute the associations observed. However, the findings were consistent with the primary analyses.

Key strengths of this study include the use of national registers, offering high-quality, prospectively recorded data with virtually complete follow-up and linkage between children and parents [21]. Parental mental disorders were identified through psychiatric hospital records, reducing the misclassification of exposure compared with self-reported symptoms. Cognitive ability was assessed using a standardized and well-validated measure administered uniformly at conscription: the Børge Priens Prøve (BPP). The BPP has demonstrated high internal reliability and substantial test–retest correlations over several decades, as well as strong correlation with the full-scale IQ of the Wechsler Adult Intelligence Scale [28], ensuring the validity of the outcome measure. The BPP test was developed in the mid-1950s, and its items have remained unchanged. Consequently, its psychometric properties may have changed over time due to shifts in population ability distributions and potential violations of measurement invariance. However, a study comparing men conscripted in 1988 and 2006–2009 found virtually identical correlations between BPP and educational level (0.52 vs. 0.53), suggesting no evidence of declining validity due to obsolescence or reduced test difficulty [26, 27, 29, 30].

This study also has some limitations. One major is unmeasured confounding. While we adjusted for several covariates to minimize confounding and improve the interpretability of the findings, genetic and shared environmental factors likely contribute to all observed associations [40–42]. As outlined in the study aim, the analyses were not intended to support causal inference, but the findings still provide relevant insight into how associations may vary across different childhood contexts. We chose not to conduct family-based designs such as within-mother sibling and maternal sibling (cousin) comparisons to account for genetic and shared environment due to limited within-family variation in mental disorders and childhood-adversities and because of concerns about reduced precision and potential selection bias of such analyses. Second, we could not determine the precise timing or chronicity of parental mental disorders or other adversities, and the temporality between adversities could not be fully assessed. As a result, it was difficult to distinguish between the factors driving the association and potential confounders. Third, selection bias may have arisen because some individuals with severe documented conditions may be exempted from examination a priori [25]. Exemption of individuals with severe mental disorders may have led to an underestimation of the observed association, as these individuals are likely to have both higher exposure to childhood adversities and lower cognitive ability. Fourth, the study included only males, and the findings adhere to males only. Females may differ in vulnerability to childhood adversities [47]. Fifth, we lacked data on specific childhood adversities (e.g., abuse and neglect) as well as factors like parenting quality/behavior, which may moderate the effect of adversities [48]. Finally, although Denmark’s universal welfare system, with free access to most health and social services, may limit the generalizability to other settings, the mechanisms linking childhood adversities to long-term outcomes are likely similar across contexts [1]. However, the strength and expression of these associations may vary across countries with different social, healthcare, and educational systems.

Although average differences in cognitive scores were modest and may be of limited clinical relevance, reducing childhood adversity may yield important population-level benefits as cognitive ability influences key life outcomes, including socioeconomic status, morbidity, and mortality [49]. While causal conclusions cannot be drawn, our findings provide relevant insight into how associations between parental mental disorders and cognitive ability vary across family contexts. These insights may also help guide future research on the role of contextual factors in shaping observed associations.

In conclusion, in this nationwide all-male Danish cohort study, having a parent diagnosed with mental disorders in hospital care before age six was associated with a slight reduction in cognitive ability at age 18 years compared to adolescents born to parents without mental disorders. However, the association was not observed among adolescents who lived with only one parent, and parental mental disorders were associated with higher cognitive scores among those placed in out-of-home care before age six. Although we cannot determine whether genetic or environmental factors underlie these patterns, the findings indicate that the association varies across family contexts. The absence of an association among adolescents who had experienced multiple additional adversities may reflect overlapping environmental, stress-related, or genetic mechanisms, whereby additional adversities do not further reduce cognitive ability beyond the association observed with parental mental disorders alone.

## Supplementary Information

Below is the link to the electronic supplementary material.

ESM 1 (DOCX 88.2 KB)

## Data Availability

The register data for this study is stored on a secure server at Statistic Denmark. Due to Danish legislation, microdata kept on the server cannot be shared for disclosure. Coding files (scripts) are available upon request by emailing the corresponding author.
