# Supplementary material for: Cognitive ability following exposure to parental mental disorders and other childhood adversities: a population-based cohort study of Danish males in late adolescence
Source: Eur J Pediatr. 2026 May 28;185(6):445. doi: 10.1007/s00431-026-07102-2 (PMC13219072; doi:10.1007/s00431-026-07102-2)
Supplement: Supplementary file 1 — (DOCX 88.2 KB) [file 431_2026_7102_MOESM1_ESM.docx]

**Appendix S1: Selected covariates for adjusted analyses of the association between parental mental disorders and cognitive ability test scores**

| **Stratifikation** | **Selected covariates included in the statistical model to adjust for confounding** |
| --- | --- |
| No stratification | Birth year, firstborn, parental highest obtained educational degree, maternal age, paternal age, family income, parental citizenship, sibling death, parental somatic illness, sibling somatic illness, sibling psychiatric illness, parental non-cohabitation |
| By number of other adversities | Birth year, firstborn, parental highest obtained educational degree, maternal age, paternal age, family income, parental citizenship |
| By Parental death | Birth year, firstborn, parental highest obtained educational degree, maternal age, paternal age, family income, parental citizenship, sibling death, parental somatic illness, sibling somatic illness, sibling psychiatric illness, parental non-cohabitation |
| By Sibling death | Birth year, firstborn, parental highest obtained educational degree, maternal age, paternal age, family income, parental citizenship, parental somatic illness, sibling somatic illness, sibling psychiatric illness, parental non-cohabitation |
| By Parental somatic illness | Birth year, firstborn, parental highest obtained educational degree, maternal age, paternal age, family income, parental citizenship, sibling death, sibling somatic illness, sibling psychiatric illness, parental non-cohabitation |
| By Sibling somatic illness | Birth year, firstborn, parental highest obtained educational degree, maternal age, paternal age, family income, parental citizenship, sibling death, parental somatic illness, sibling psychiatric illness, parental non-cohabitation |
| By Sibling psychiatric illness | Birth year, firstborn, parental highest obtained educational degree, maternal age, paternal age, family income, parental citizenship, sibling death, parental somatic illness, sibling somatic illness, parental non-cohabitation |
| By Poverty | Birth year, firstborn, parental highest obtained educational degree, maternal age, paternal age, parental citizenship, sibling death, parental somatic illness, sibling somatic illness, sibling psychiatric illness, parental non-cohabitation |
| By Parental long-term unemployment | Birth year, firstborn, parental highest obtained educational degree, maternal age, paternal age, family income, parental citizenship, sibling death, parental somatic illness, sibling somatic illness, sibling psychiatric illness, parental non-cohabitation |
| By Parental non-cohabitation | Birth year, firstborn, parental highest obtained educational degree, maternal age, paternal age, family income, parental citizenship, sibling death, parental somatic illness, sibling somatic illness, sibling psychiatric illness |
| By Inter-municipal migrations | Birth year, firstborn, parental highest obtained educational degree, maternal age, paternal age, family income, parental citizenship, sibling death, parental somatic illness, sibling somatic illness, sibling psychiatric illness, parental non-cohabitation |
| By Placement | Birth year, firstborn, parental highest obtained educational degree, maternal age, paternal age, family income, parental citizenship, sibling death, parental somatic illness, sibling somatic illness, sibling psychiatric illness, parental non-cohabitation |

**Table S1, Associations between parental mental disorders in the first six years of life and cognitive ability test scores in males at age 18 years, overall and stratified by other childhood adversities**

|  | | **Parental mental disorder** | | | | **Association between parental mental disorders and cognitive ability test scores** | | |
| --- | --- | --- | --- | --- | --- | --- | --- | --- |
|  | | **No** | | **Yes** | | **Crude mean difference**  **(95% CI)** | **Adj. mean difference**  **(95% CI)** | **p-value effect modification** |
|  |  | **n** | **Mean score**  **(95% CI)** | **n** | **Mean score**  **(95% CI)** |  |  |  |
|  | All | 119.083 | 100 (100;100) | 6708 | 96 (96;96) | -4.16 (-4.55;-3.77) | -0.91 (-1.28;-0.53) |  |
| **By other health-related adversity** | |  |  |  |  |  |  |  |
| Parental death | No | 118.517 | 100 (100;100) | 6585 | 96 (96;96) | -4.17 (-4.56;-3.77) | -0.92 (-1.29;-0.54) | 0.338 |
|  | Yes | 566 | 97 (96;98) | 123 | 96 (93;99) | -1.20 (-4.47;2.07) | 0.56 (-2.44;3.57) |  |
| Sibling death | No | 118.629 | 100 (100;100) | 6660 | 96 (96;97) | -4.15 (-4.54;-3.75) | -0.91 (-1.28;-0.53) | 0.921 |
|  | Yes | 454 | 97 (95;98) | 48 | 93 (88;98) | -3.86 (-8.82;1.11) | -0.68 (-5.21;3.85) |  |
| Parental somatic illness | No | 113.345 | 100 (100;100) | 6157 | 96 (96;97) | -4.11 (-4.52;-3.71) | -0.92 (-1.31;-0.53) | 0.813 |
|  | Yes | 5738 | 98 (98;99) | 551 | 95 (93;96) | -3.85 (-5.21;-2.49) | -0.76 (-2.05;0.54) |  |
| Sibling somatic illness | No | 114.967 | 100 (100;100) | 6435 | 96 (96;97) | -4.12 (-4.51;-3.72) | -0.89 (-1.27;-0.51) | 0.661 |
|  | Yes | 4116 | 98 (98;99) | 273 | 93 (92;95) | -4.77 (-6.70;-2.84) | -1.30 (-3.08;0.49) |  |
| Sibling mental dirsorders | No | 116.488 | 100 (100;100) | 6352 | 96 (96;97) | -4.12 (-4.52;-3.72) | -0.95 (-1.33;-0.57) | 0.319 |
|  | Yes | 2595 | 97 (96;97) | 356 | 94 (92;96) | -2.49 (-4.23;-0.74) | -0.08 (-1.75;1.59) |  |
| **By socioeconomic disadvantage** | |  |  |  |  |  |  |  |
| Poverty | No | 93.878 | 102 (102;102) | 3617 | 99 (98;99) | -2.74 (-3.24;-2.24) | -1.10 (-1.58;-0.63) | 0.085 |
|  | Yes | 24.217 | 95 (94;95) | 3026 | 93 (92;93) | -1.87 (-2.47;-1.26) | -0.45 (-1.03;0.13) |  |
| Parental long-term unemployment* | No | 4.9153 | 102 (102;102) | 1548 | 100 (100;101) | -1.79 (-2.52;-1.06) | -0.51 (-1.21;0.19) | 0.906 |
|  | Yes | 21.488 | 96 (96;96) | 2601 | 93 (93;94) | -2.43 (-3.08;-1.78) | -0.56 (-1.19;0.06) |  |
| **By family instability** | |  |  |  |  |  |  |  |
| Parental non-cohabitation | No | 93.429 | 101 (101;101) | 2853 | 98 (97;98) | -3.51 (-4.12;-2.91) | -1.58 (-2.14;-1.03) | **0.001** |
|  | Yes | 25.654 | 97 (97;97) | 3855 | 95 (94;95) | -1.86 (-2.38;-1.33) | -0.35 (-0.84;0.15) |  |
| ≥3 inter-municipal migrations | No | 116.111 | 100 (100;100) | 6328 | 96 (96;97) | -4.12 (-4.52;-3.72) | -0.90 (-1.28;-0.51) | 0.469 |
|  | Yes | 2972 | 101 (100;101) | 380 | 96 (94;97) | -5.09 (-6.69;-3.49) | -1.47 (-2.98;0.04) |  |
| Placement | No | 118.726 | 100 (100;100) | 6445 | 96 (96;97) | -4.02 (-4.41;-3.62) | -0.93 (-1.31;-0.56) | **0.002** |
|  | yes | 357 | 89 (87;91) | 263 | 92 (90;94) | 2.95 (0.31;5.59) | 3.18 (0.59;5.76) |  |
| **By number of other childhood adversities** | 0 | 60.708 | 103 (103;103) | 1223 | 102 (101;103) | -1.06 (-1.87;-0.25) | -0.66 (-1.45;0.13) | **0.004** |
|  | 1 | 32.130 | 100 (100;100) | 1530 | 98 (98;99) | -1.86 (-2.65;-1.07) | -1.52 (-2.27;-0.77) |  |
|  | 2 | 15.936 | 96 (95;96) | 1684 | 94 (94;95) | -1.13 (-1.92;-0.34) | -1.06 (-1.80;-0.32) |  |
|  | 3 | 8578 | 93 (93;93) | 1681 | 93 (93;94) | 0.14 (-0.68;0.96) | 0.27 (-0.52;1.07) |  |
|  | ≥4 | 1731 | 91 (90;92) | 590 | 92 (90;93) | 0.55 (-0.93;2.02) | 0.73 (-0.70;2.17) |  |

Abbreviations: Adj.=adjusted, 95% CI=95% confidence interval.

*The analysis of long-term unemployment was restricted to individuals born between 1998 and 2001.

Estimates are presented as mean differences derived from linear regression analyses. Covariates in the adjusted model for the overall association were birth year, firstborn status, parental highest educational level, maternal age, paternal age, family income, parental citizenship, sibling death, parental somatic illness, sibling somatic illness, sibling psychiatric illness, and parental non-cohabitation. Appendix S1 describes the covariates included in models assessing associations within stratum of childhood adversities.

To assess effect modification, the models included an interaction term between parental mental disorders and the selected childhood adversity and the variables main effects. The p-value is obtained using the Wald test.

**Table S2, Associations between individual childhood adversities in the first six years of life and cognitive ability test scores in males at age 18 years, overall and stratified by parental mental disorders**

|  | | **Childhood adversity** | | | | **Association between childhood adversity and cognitive ability test scores** | | |
| --- | --- | --- | --- | --- | --- | --- | --- | --- |
|  | | **No** | | **Yes** | | **Crude mean difference**  **(95% CI)** | **Adj. mean difference**  **(95% CI)** | **p-value effect modification** |
|  |  | **n** | **Mean score**  **(95% CI)** | **n** | **Mean score**  **(95% CI)** |  |  |  |
| **Parental death** | | | | | | | | |
|  | All | 125.102 | 100 (100;100) | 689 | 97 (96;98) | -3.23 (-4.49;-1.98) | -1.19 (-2.35;-0.02) |  |
| By parental mental disorders | No | 118.517 | 100 (100;100) | 566 | 97 (96;98) | -3.24 (-4.62;-1.86) | -1.45 (-2.73;-0.17) | 0.338 |
|  | Yes | 6585 | 96 (96;96) | 123 | 96 (93;99) | -0.27 (-3.26;2.72) | 0.03 (-2.72;2.78) |  |
| **Sibling death** | | | | | | | | |
|  | All | 125.289 | 100 (100;100) | 502 | 96 (95;98) | -3.76 (-5.31;-2.21) | -2.09 (-3.52;-0.66) |  |
| By parental mental disorders | No | 118.629 | 100 (100;100) | 454 | 97 (95;98) | -3.61 (-5.25;-1.97) | -2.11 (-3.62;-0.60) | 0.921 |
|  | Yes | 6660 | 96 (96;97) | ***48*** | 93 (88;98) | -3.32 (-8.02;1.38) | -1.88 (-6.17;2.41) |  |
| **Parental somatic illness** | | | | | | | | |
|  | All | 119.502 | 100 (100;100) | 6289 | 98 (98;98) | -2.03 (-2.44;-1.63) | -0.90 (-1.27;-0.53) |  |
| By parental mental disorders | No | 113.345 | 100 (100;100) | 5738 | 98 (98;99) | -1.91 (-2.33;-1.49) | -0.91 (-1.30;-0.53) | 0.813 |
|  | Yes | 6157 | 96 (96;97) | 551 | 95 (93;96) | -1.65 (-3.00;-0.29) | -0.75 (-2.03;0.54) |  |
| **Sibling somatic illness** | | | | | | | | |
|  | All | 121.402 | 100 (100;100) | 4389 | 98 (97;98) | -2.19 (-2.68;-1.70) | -0.72 (-1.17;-0.27) |  |
| By parental mental disorders | No | 114.967 | 100 (100;100) | 4116 | 98 (98;99) | -2.11 (-2.61;-1.61) | -0.70 (-1.16;-0.24) | 0.661 |
|  | Yes | 6435 | 96 (96;97) | 273 | 93 (92;95) | -2.77 (-4.67;-0.86) | -1.11 (-2.87;0.66) |  |
| **Sibling mental disorders** | | | | | | | | |
|  | All | 122.840 | 100 (100;100) | 2951 | 96 (96;97) | -3.92 (-4.51;-3.33) | -1.75 (-2.31;-1.19) |  |
| By parental mental disorders | No | 116.488 | 100 (100;100) | 2595 | 97 (96;97) | -3.83 (-4.46;-3.20) | -1.85 (-2.45;-1.26) | 0.319 |
|  | Yes | 6352 | 96 (96;97) | 356 | 94 (92;96) | -2.19 (-3.87;-0.52) | -0.98 (-2.59;0.62) |  |
| **Poverty** | | | | | | | | |
|  | All | 97.495 | 102 (102;102) | 27243 | 94 (94;95) | -7.10 (-7.32;-6.88) | -1.76 (-2.00;-1.52) |  |
| By parental mental disorders | No | 93.878 | 102 (102;102) | 24217 | 95 (94;95) | -7.00 (-7.23;-6.77) | -1.82 (-2.06;-1.57) | 0.085 |
|  | Yes | 3617 | 99 (98;99) | 3026 | 93 (92;93) | -6.12 (-6.87;-5.37) | -1.16 (-1.88;-0.43) |  |
| **Parental long-term unemployment*** | | | | | | | | |
| By parental mental disorders | All  No | 50.701  49.153 | 102 (102;102)  102 (102;102) | 24089  21488 | 96 (95;96)  96 (96;96) | -6.42 (-6.65;-6.18)  -6.21 (-6.46;-5.96) | -1.90 (-2.15;-1.65)  -1.90 (-2.16;-1.64) | 0.906 |
|  | Yes | 1548 | 100 (100;101) | 2601 | 93 (93;94) | -6.85 (-7.79;-5.91) | -1.95 (-2.86;-1.04) |  |
| **Parental non-cohabitation** | | | | | | | | |
|  | All | 96282 | 101 (101;101) | 29509 | 97 (96;97) | -4.58 (-4.78;-4.37) | -2.05 (-2.25;-1.84) |  |
| By parental mental disorders | No | 93429 | 101 (101;101) | 25654 | 97 (97;97) | -4.44 (-4.65;-4.22) | -2.14 (-2.35;-1.93) | **0.001** |
|  | Yes | 2853 | 98 (97;98) | 3855 | 95 (94;95) | -2.78 (-3.55;-2.01) | -0.90 (-1.62;-0.18) |  |
| **≥3 inter-municipal migrations** | | | | | | | | |
|  | All | 122439 | 100 (100;100) | 3352 | 100 (100;101) | 0.29 (-0.24;0.83) | 1.64 (1.14;2.14) |  |
| By parental mental disorders | No | 116111 | 100 (100;100) | 2972 | 101 (100;101) | 0.66 (0.09;1.22) | 1.70 (1.18;2.23) | 0.469 |
|  | Yes | 6328 | 96 (96;97) | 380 | 96 (94;97) | -0.31 (-1.86;1.24) | 1.13 (-0.34;2.59) |  |
| **Placement** | | | | | | | | |
|  | All | 125171 | 100 (100;100) | 620 | 90 (89;91) | -9.96 (-11.29;-8.63) | -2.75 (-4.06;-1.43) |  |
| By parental mental disorders | No | 118726 | 100 (100;100) | 357 | 89 (87;91) | -11.42 (-13.20;-9.64) | -4.46 (-6.18;-2.74) | **0.002** |
|  | Yes | 6445 | 96 (96;97) | 263 | 92 (90;94) | -4.45 (-6.45;-2.46) | -0.35 (-2.33;1.63) |  |

Abbreviations: Adj.=adjusted, 95% CI=95% confidence interval.

*The analysis of long-term unemployment was restricted to individuals born between 1998 and 2001.

Estimates are presented as mean differences derived from linear regression analyses. Covariates in the adjusted models correspond to the stratified analyses, as specified in appendix S1. To assess effect modification, the models included an interaction term between parental mental disorders and the selected childhood adversity and the variables main effects. The p-value is obtained using the Wald test.

**Table S3, Associations between the number of other childhood adversities in the first six years and cognitive ability test scores in males at age 18 years, stratified by parental mental disorders**

| **Parental psyciatric disorders** | **Number of other childhood adversities** | | | **Association between childhood adversity and cognitive ability test scores** | | |
| --- | --- | --- | --- | --- | --- | --- |
|  |  | **n** | **Mean score**  **(95% CI)** | **Crude mean difference**  **(95% CI)** | **Adj. mean difference**  **(95% CI)** | **p-value effect modification** |
| No | 0 | 60.708 | 103 (103;103) | ref. | ref. | **0.004** |
| No | 1 | 32.130 | 100 (100;100) | -2.56 (-2.76;-2.36) | -0.97 (-1.16;-0.77) |  |
| No | 2 | 15.936 | 96 (95;96) | -7.20 (-7.47;-6.92) | -2.89 (-3.18;-2.61) |  |
| No | 3 | 8578 | 93 (93;93) | -9.69 (-10.05;-9.33) | -4.15 (-4.53;-3.76) |  |
| No | ≥4 | 1731 | 91 (90;92) | -11.84 (-12.63;-11.05) | -5.91 (-6.69;-5.13) |  |
| Yes | 0 | 1223 | 102 (101;103) | ref. | ref. |  |
| Yes | 1 | 1530 | 98 (98;99) | -3.36 (-4.47;-2.25) | -1.83 (-2.90;-0.76) |  |
| Yes | 2 | 1684 | 94 (94;95) | -7.27 (-8.37;-6.17) | -3.29 (-4.35;-2.24) |  |
| Yes | 3 | 1681 | 93 (93;94) | -8.49 (-9.59;-7.40) | -3.21 (-4.29;-2.13) |  |
| Yes | ≥4 | 590 | 92 (90;93) | -10.23 (-11.73;-8.74) | -4.51 (-5.99;-3.04) |  |

Abbreviations: Adj.=adjusted, 95% CI=95% confidence interval.

Estimates are presented as mean differences derived from linear regression analyses.

Covariates in the adjusted model included birth year, firstborn, parental highest obtained educational degree, maternal age, paternal age, family income, and parental citizenship. To assess effect modification, the models included an interaction term between parental mental disorders and the selected childhood adversity and the variables main effects. The p-value is obtained using the Wald test.

**Table S4, Associations between parental mental disorders in the first six years of life and cognitive ability test scores in males at age 18 years, according to whether the child lived with the affected parent or the affected parent died**

| **Parental menal disorders** | **Living situation in raltion the parent with a mental disorder** | **n** | **Mean score**  **(95% CI)** | **Crude mean difference**  **(95% CI)** | **Adj. mean difference**  **(95% CI)** |
| --- | --- | --- | --- | --- | --- |
| No |  | 119083 | 100 (100;100) | ref. | ref. |
| Yes | Child not co-habiting the affected parent | 4178 | 97 (96;97) | -3.42 (-3.91;-2.92) | -1.37 (-1.83;-0.91) |
|  | Child co-habiting the affected parent | 2530 | 95 (94;95) | -5.37 (-5.98;-4.76) | -1.67 (-2.26;-1.08) |
|  | Affected parent alive | 6629 | 96 (96;97) | -4.14 (-4.53;-3.75) | -0.90 (-1.28;-0.53) |
|  | Affected parent deceased | 79 | 95 (91;99) | -5.28 (-9.15;-1.40) | -1.17 (-4.72;2.38) |

Abbreviations: Adj.=adjusted, 95% CI=95% confidence interval. Estimates are presented as mean differences derived from linear regression analyses. Covariates in the adjusted model were birth year, firstborn status, parental highest educational level, maternal age, paternal age, family income, parental citizenship, sibling death, parental somatic illness, sibling somatic illness, and sibling psychiatric illness. In analyses of parental death, the model additionally included parental non-cohabitation.

**Table S5, Associations between parental mental disorders in the first 12 years of life and cognitive ability test scores in males at age 18 years, overall and stratified by other childhood adversities**

|  | | **Parental mental disorder** | | | | **Association between parental mental disorders and cognitive ability test scores** | | |
| --- | --- | --- | --- | --- | --- | --- | --- | --- |
|  | | **No** | | **Yes** | | **Crude mean difference**  **(95% CI)** | **Adj. mean difference**  **(95% CI)** | **p-value effect modification** |
|  |  | **n** | **Mean score**  **(95% CI)** | **n** | **Mean score**  **(95% CI)** |  |  |  |
|  | All | 113019 | 100 (100;101) | 12772 | 96 (96;96) | -4.39 (-4.69;-4.10) | -1.00 (-1.28;-0.72) |  |
| **By other health-related adversity** | |  |  |  |  |  |  |  |
| Parental death | No | 111534 | 101 (100;101) | 12257 | 96 (96;96) | -4.36 (-4.66;-4.06) | -0.97 (-1.26;-0.68) | 0.852 |
|  | Yes | 1485 | 97 (97;98) | 515 | 94 (93;96) | -3.13 (-4.77;-1.50) | -0.82 (-2.35;0.70) |  |
| Sibling death | No | 112284 | 101 (100;101) | 12622 | 96 (96;96) | -4.38 (-4.67;-4.08) | -1.00 (-1.29;-0.72) | 0.879 |
|  | Yes | 735 | 96 (95;97) | 150 | 92 (90;95) | -3.59 (-6.64;-0.54) | -0.78 (-3.58;2.02) |  |
| Parental somatic illness | No | 104352 | 101 (101;101) | 11251 | 96 (96;97) | -4.28 (-4.59;-3.97) | -0.97 (-1.27;-0.67) | 0.541 |
|  | Yes | 8667 | 99 (99;99) | 1521 | 94 (94;95) | -4.71 (-5.59;-3.82) | -1.24 (-2.07;-0.41) |  |
| Sibling somatic illness | No | 107556 | 101 (101;101) | 11940 | 96 (96;97) | -4.31 (-4.61;-4.00) | -0.96 (-1.25;-0.67) | 0.264 |
|  | Yes | 5463 | 99 (98;99) | 832 | 93 (92;95) | -5.09 (-6.28;-3.89) | -1.60 (-2.70;-0.51) |  |
| Sibling mental dirsorders | No | 105710 | 101 (101;101) | 10964 | 96 (96;97) | -4.24 (-4.55;-3.92) | -1.05 (-1.35;-0.75) | 0.355 |
|  | Yes | 7309 | 97 (97;97) | 1808 | 94 (93;94) | -3.37 (-4.20;-2.54) | -0.66 (-1.44;0.13) |  |
| **By socioeconomic disadvantage** | |  |  |  |  |  |  |  |
| Poverty | No | 84419 | 102 (102;102) | 5933 | 100 (99;100) | -2.44 (-2.83;-2.05) | -0.97 (-1.34;-0.59) | 0.536 |
|  | Yes | 28600 | 96 (95;96) | 6839 | 93 (93;93) | -2.63 (-3.06;-2.20) | -0.79 (-1.21;-0.38) |  |
| Parental long-term unemployment* | No | 42902 | 103 (102;103) | 2224 | 101 (100;101) | -1.68 (-2.29;-1.07) | -0.53 (-1.11;0.06) | 0.807 |
|  | Yes | 24089 | 97 (97;97) | 5575 | 94 (94;94) | -2.75 (-3.22;-2.28) | -0.62 (-1.07;-0.17) |  |
| **By family instability** | |  |  |  |  |  |  |  |
| Parental non-cohabitation | No | 78162 | 102 (102;102) | 4307 | 98 (98;99) | -3.57 (-4.07;-3.08) | -1.38 (-1.84;-0.93) | **0.036** |
|  | Yes | 34857 | 98 (98;98) | 8465 | 95 (95;95) | -2.84 (-3.21;-2.46) | -0.77 (-1.13;-0.41) |  |
| ≥3 inter-municipal migrations | No | 107623 | 101 (100;101) | 11388 | 96 (96;97) | -4.31 (-4.62;-4.00) | -0.97 (-1.27;-0.67) | 0.276 |
|  | Yes | 5396 | 100 (100;100) | 1384 | 95 (94;96) | -4.78 (-5.73;-3.82) | -1.49 (-2.38;-0.60) |  |
| Placement | No | 112462 | 101 (100;101) | 12096 | 96 (96;97) | -4.07 (-4.37;-3.78) | -0.92 (-1.20;-0.63) | **0.030** |
|  | yes | 557 | 89 (88;90) | 676 | 89 (88;91) | 0.45 (-1.39;2.28) | 1.10 (-0.70;2.91) |  |
| **By number of other childhood adversities** | 0 | 43864 | 103 (103;104) | 1301 | 103 (102;103) | -0.86 (-1.66;-0.06) | -0.70 (-1.47;0.08) | 0.562 |
|  | 1 | 33692 | 101 (101;101) | 2444 | 100 (100;101) | -1.09 (-1.67;-0.51) | -0.90 (-1.46;-0.34) |  |
|  | 2 | 19431 | 98 (97;98) | 3045 | 96 (96;97) | -1.30 (-1.91;-0.69) | -0.77 (-1.34;-0.20) |  |
|  | 3 | 11626 | 95 (95;95) | 3593 | 94 (93;94) | -1.48 (-2.08;-0.88) | -0.69 (-1.26;-0.11) |  |
|  | ≥4 | 4406 | 93 (92;93) | 2389 | 92 (91;93) | -0.54 (-1.35;0.27) | -0.08 (-0.86;0.71) |  |

Abbreviations: Adj.=adjusted, 95% CI=95% confidence interval.

*The analysis of long-term unemployment was restricted to individuals born between 1998 and 2001.

Estimates are presented as mean differences derived from linear regression analyses. To assess effect modification, the models included an interaction term between parental mental disorders and the selected childhood adversity and the variables main effects. The p-value is obtained using the Wald test.

**Table S6, Associations between parental anxiety or depression in the first six years of life and cognitive ability test scores in males at age 18 years, overall and stratified by other childhood adversities**

|  | | **Parental mental disorder** | | | | **Association between parental mental disorders and cognitive ability test scores** | | |
| --- | --- | --- | --- | --- | --- | --- | --- | --- |
|  | | **No** | | **Yes** | | **Crude mean difference**  **(95% CI)** | **Adj. mean difference**  **(95% CI)** | **p-value effect modification** |
|  |  | **n** | **Mean score**  **(95% CI)** | **n** | **Mean score**  **(95% CI)** |  |  |  |
|  | All | 120544 | 100 (100;100) | 5247 | 96 (96;97) | -3.91 (-4.35;-3.47) | 0.22 (-0.24;0.68) |  |
| **By other health-related adversity** | |  |  |  |  |  |  |  |
| Parental death | No | 119946 | 100 (100;100) | 5156 | 96 (96;97) | -3.94 (-4.37;-3.50) | 0.20 (-0.26;0.66) | 0.303 |
|  | Yes | 598 | 97 (96;98) | 91 | 97 (93;100) | -0.02 (-3.68;3.63) | 1.98 (-1.38;5.34) |  |
| Sibling death | No | 120080 | 100 (100;100) | 5209 | 96 (96;97) | -3.90 (-4.34;-3.46) | 0.22 (-0.24;0.68) | 0.936 |
|  | Yes | 464 | 97 (95;98) | 38 | 93 (88;98) | -3.58 (-8.88;1.72) | 0.03 (-4.70;4.75) |  |
| Parental somatic illness | No | 114680 | 100 (100;100) | 4822 | 96 (96;97) | -3.85 (-4.30;-3.39) | 0.24 (-0.24;0.71) | 0.731 |
|  | Yes | 5864 | 98 (98;99) | 425 | 95 (93;96) | -3.83 (-5.35;-2.31) | -0.03 (-1.49;1.43) |  |
| Sibling somatic illness | No | 116353 | 100 (100;100) | 5049 | 96 (96;97) | -3.92 (-4.36;-3.47) | 0.20 (-0.27;0.67) | 0.660 |
|  | Yes | 4191 | 98 (98;99) | 198 | 95 (92;97) | -3.59 (-5.81;-1.36) | 0.67 (-1.39;2.72) |  |
| Sibling mental dirsorders | No | 117864 | 100 (100;100) | 4976 | 96 (96;97) | -3.87 (-4.32;-3.43) | -0.06 (-0.53;0.41) | 0.579 |
|  | Yes | 2680 | 96 (96;97) | 271 | 94 (92;96) | -2.38 (-4.36;-0.40) | 0.55 (-1.32;2.43) |  |
| **By socioeconomic disadvantage** | |  |  |  |  |  |  |  |
| Poverty | No | 94557 | 102 (102;102) | 2938 | 99 (98;100) | -2.66 (-3.21;-2.10) | 0.07 (-0.50;0.64) | 0.330 |
|  | Yes | 24984 | 95 (94;95) | 2259 | 93 (92;94) | -1.73 (-2.42;-1.05) | 0.49 (-0.20;1.17) |  |
| Parental long-term unemployment* | No | 49379 | 102 (102;102) | 1322 | 100 (100;101) | -1.73 (-2.52;-0.93) | 0.34 (-0.48;1.16) | 0.626 |
|  | Yes | 22078 | 96 (96;96) | 2011 | 94 (93;94) | -2.24 (-2.97;-1.51) | 0.08 (-0.65;0.82) |  |
| **By family instability** | |  |  |  |  |  |  |  |
| Parental non-cohabitation | No | 93949 | 101 (101;101) | 2333 | 98 (97;98) | -3.60 (-4.27;-2.94) | -0.54 (-1.18;0.11) | **0.001** |
|  | Yes | 26595 | 97 (97;97) | 2914 | 95 (95;96) | -1.43 (-2.01;-0.84) | 0.84 (0.25;1.43) |  |
| ≥3 inter-municipal migrations | No | 117475 | 100 (100;100) | 4964 | 96 (96;97) | -3.88 (-4.33;-3.43) | 0.25 (-0.22;0.73) | 0.298 |
|  | Yes | 3069 | 101 (100;101) | 283 | 96 (94;98) | -4.70 (-6.52;-2.88) | -0.69 (-2.43;1.05) |  |
| Placement | No | 120104 | 100 (100;100) | 5067 | 96 (96;97) | -3.78 (-4.22;-3.34) | 0.18 (-0.29;0.65) | **0.046** |
|  | yes | 440 | 90 (88;91) | 180 | 92 (89;94) | 2.01 (-0.85;4.87) | 3.08 (0.26;5.89) |  |
| **By number of other childhood adversities** | 0 | 60911 | 103 (103;103) | 1020 | 101 (101;102) | -1.32 (-2.20;-0.44) | 0.21 (-0.68;1.10) | **0.016** |
|  | 1 | 32417 | 100 (100;100) | 1243 | 98 (98;99) | -1.74 (-2.63;-0.86) | -0.50 (-1.37;0.36) |  |
|  | 2 | 16278 | 96 (95;96) | 1342 | 94 (94;95) | -1.23 (-2.11;-0.35) | -0.27 (-1.10;0.57) |  |
|  | 3 | 9013 | 93 (93;93) | 1246 | 93 (93;94) | 0.34 (-0.58;1.25) | 1.28 (0.37;2.19) |  |
|  | ≥4 | 1925 | 91 (90;92) | 396 | 92 (90;93) | 0.90 (-0.81;2.61) | 1.57 (-0.11;3.25) |  |

Abbreviations: Adj.=adjusted, 95% CI=95% confidence interval.

*The analysis of long-term unemployment was restricted to individuals born between 1998 and 2001.

Estimates are presented as mean differences derived from linear regression analyses. Covariates in the adjusted model for the overall association were birth year, firstborn status, parental highest educational level, maternal age, paternal age, family income, parental citizenship, sibling death, parental somatic illness, sibling somatic illness, sibling psychiatric illness, and parental non-cohabitation. Appendix S1 describes the covariates included in models assessing associations within stratum of childhood adversities. Additionally, the analyses were adjusted for other parental mental disorders than anxiety/depression.

To assess effect modification, the models included an interaction term between parental mental disorders and the selected childhood adversity and the variables main effects. The p-value is obtained using the Wald test.

**Table S7, Associations between parental bipolar disorders in the first six years of life and cognitive ability test scores in males at age 18 years, overall and stratified by other childhood adversities**

|  | | **Parental mental disorder** | | | | **Association between parental mental disorders and cognitive ability test scores** | | |
| --- | --- | --- | --- | --- | --- | --- | --- | --- |
|  | | **No** | | **Yes** | | **Crude mean difference**  **(95% CI)** | **Adj. mean difference**  **(95% CI)** | **p-value effect modification** |
|  |  | **n** | **Mean score**  **(95% CI)** | **n** | **Mean score**  **(95% CI)** |  |  |  |
|  | All | 124441 | 100 (100;100) | 1350 | 99 (99;100) | -0.70 (-1.50;0.10) | 2.02 (0.75;3.28) |  |
| **By other health-related adversity** | |  |  |  |  |  |  |  |
| Parental death | No | 123762 | 100 (100;100) | 1340 | 99 (99;100) | -0.69 (-1.50;0.11) | 2.06 (0.79;3.33) | 0.427 |
|  | Yes | 679 | 97 (96;98) | 10 | 97 (84;109) | -0.22 (-10.60;10.16) | -1.55 (-10.38;7.27) |  |
| Sibling death | No | 123944 | 100 (100;100) | 1345 | 99 (99;100) | -0.72 (-1.52;0.08) | 2.05 (0.78;3.31) | **0.000** |
|  | Yes | 497 | 96 (95;98) | 5 | 101 (73;129) | 4.84 (-12.77;22.45) | -11.66 (-16.32;-7.01) |  |
| Parental somatic illness | No | 118243 | 100 (100;100) | 1259 | 99 (99;100) | -0.74 (-1.57;0.09) | 2.03 (0.72;3.34) | 0.929 |
|  | Yes | 6198 | 98 (98;98) | 91 | 99 (96;101) | 0.46 (-2.41;3.34) | 1.82 (-2.79;6.42) |  |
| Sibling somatic illness | No | 120117 | 100 (100;100) | 1285 | 99 (99;100) | -0.83 (-1.66;0.00) | 1.77 (0.47;3.07) | **0.041** |
|  | Yes | 4324 | 98 (97;98) | 65 | 100 (98;103) | 2.51 (-0.32;5.33) | 6.91 (2.15;11.67) |  |
| Sibling mental dirsorders | No | 121565 | 100 (100;100) | 1275 | 99 (99;100) | -0.73 (-1.56;0.09) | 1.90 (0.58;3.22) | 0.756 |
|  | Yes | 2876 | 96 (96;97) | 75 | 98 (95;102) | 2.24 (-0.92;5.40) | 2.82 (-1.74;7.39) |  |
| **By socioeconomic disadvantage** | |  |  |  |  |  |  |  |
| Poverty | No | 96621 | 102 (102;102) | 874 | 101 (100;102) | -0.36 (-1.32;0.61) | 1.66 (0.06;3.25) | 0.556 |
|  | Yes | 26779 | 94 (94;95) | 464 | 96 (95;97) | 1.53 (0.16;2.89) | 2.43 (0.38;4.49) |  |
| Parental long-term unemployment* | No | 50339 | 102 (102;102) | 362 | 102 (101;103) | -0.06 (-1.47;1.35) | 1.81 (-0.57;4.19) | 0.783 |
|  | Yes | 23673 | 96 (95;96) | 416 | 97 (96;99) | 1.72 (0.30;3.14) | 2.25 (0.17;4.34) |  |
| **By family instability** | |  |  |  |  |  |  |  |
| Parental non-cohabitation | No | 95595 | 101 (101;101) | 687 | 102 (101;103) | 0.45 (-0.64;1.54) | 2.66 (0.66;4.66) | 0.416 |
|  | Yes | 28846 | 97 (96;97) | 663 | 97 (96;98) | 0.53 (-0.61;1.67) | 1.60 (-0.02;3.21) |  |
| ≥3 inter-municipal migrations | No | 121169 | 100 (100;100) | 1270 | 99 (98;100) | -0.83 (-1.66;0.00) | 1.72 (0.41;3.03) | 0.061 |
|  | Yes | 3272 | 100 (100;101) | 80 | 102 (99;104) | 1.31 (-1.41;4.02) | 6.18 (1.72;10.63) |  |
| Placement | No | 123860 | 100 (100;100) | 1311 | 100 (99;100) | -0.51 (-1.32;0.30) | 2.35 (1.05;3.64) | **0.052** |
|  | yes | 581 | 90 (89;91) | 39 | 92 (87;96) | 1.52 (-2.55;5.59) | -2.92 (-8.09;2.24) |  |
| **By number of other childhood adversities** | 0 | 61575 | 103 (103;103) | 356 | 103 (101;104) | 0.06 (-1.47;1.59) | 1.46 (-1.79;4.70) | 0.838 |
|  | 1 | 33318 | 100 (100;100) | 342 | 101 (100;103) | 1.08 (-0.41;2.57) | 1.80 (-0.65;4.25) |  |
|  | 2 | 17314 | 95 (95;96) | 306 | 98 (96;99) | 2.29 (0.80;3.79) | 2.74 (0.56;4.93) |  |
|  | 3 | 10001 | 93 (93;93) | 258 | 96 (94;97) | 2.45 (0.44;4.46) | 2.91 (0.04;5.78) |  |
|  | ≥4 | 2233 | 91 (90;92) | 88 | 95 (92;98) | 4.04 (1.20;6.87) | 0.51 (-3.58;4.60) |  |

Abbreviations: Adj.=adjusted, 95% CI=95% confidence interval.

*The analysis of long-term unemployment was restricted to individuals born between 1998 and 2001.

Estimates are presented as mean differences derived from linear regression analyses. Covariates in the adjusted model for the overall association were birth year, firstborn status, parental highest educational level, maternal age, paternal age, family income, parental citizenship, sibling death, parental somatic illness, sibling somatic illness, sibling psychiatric illness, and parental non-cohabitation. Appendix S1 describes the covariates included in models assessing associations within stratum of childhood adversities. Additionally, the analyses were adjusted for other parental mental disorders than bipolar disorders. To assess effect modification, the models included an interaction term between parental mental disorders and the selected childhood adversity and the variables main effects. The p-value is obtained using the Wald test.

**Table S8, Associations between parental schizophrenia in the first six years of life and cognitive ability test scores in males at age 18 years, overall and stratified by other childhood adversities**

|  | | **Parental mental disorder** | | | | **Association between parental mental disorders and cognitive ability test scores** | | |
| --- | --- | --- | --- | --- | --- | --- | --- | --- |
|  | | **No** | | **Yes** | | **Crude mean difference**  **(95% CI)** | **Adj. mean difference**  **(95% CI)** | **p-value effect modification** |
|  |  | **n** | **Mean score**  **(95% CI)** | **n** | **Mean score**  **(95% CI)** |  |  |  |
|  | All | 123785 | 100 (100;100) | 2006 | 94 (93;94) | -6.49 (-7.23;-5.75) | -1.31 (-2.01;-0.62) |  |
| **By other health-related adversity** | |  |  |  |  |  |  |  |
| Parental death | No | 123122 | 100 (100;100) | 1980 | 94 (93;94) | -6.44 (-7.18;-5.70) | -1.26 (-1.96;-0.56) | 0.205 |
|  | Yes | 663 | 97 (96;98) | 26 | 89 (81;96) | -8.46 (-15.59;-1.33) | -5.40 (-11.77;0.97) |  |
| Sibling death | No | 123298 | 100 (100;100) | 1991 | 94 (93;94) | -6.44 (-7.17;-5.70) | -1.26 (-1.96;-0.56) | 0.128 |
|  | Yes | 487 | 97 (95;98) | 15 | 84 (76;93) | -12.23 (-20.80;-3.66) | -8.60 (-18.03;0.83) |  |
| Parental somatic illness | No | 117661 | 100 (100;100) | 1841 | 94 (93;95) | -6.35 (-7.11;-5.58) | -1.25 (-1.97;-0.53) | 0.553 |
|  | Yes | 6124 | 98 (98;99) | 165 | 91 (88;94) | -7.35 (-9.92;-4.78) | -2.04 (-4.57;0.49) |  |
| Sibling somatic illness | No | 119496 | 100 (100;100) | 1906 | 94 (93;94) | -6.46 (-7.22;-5.71) | -1.33 (-2.04;-0.62) | 0.828 |
|  | Yes | 4289 | 98 (98;99) | 100 | 92 (89;95) | -6.38 (-9.60;-3.15) | -0.97 (-4.19;2.26) |  |
| Sibling mental dirsorders | No | 120954 | 100 (100;100) | 1886 | 94 (93;95) | -6.44 (-7.20;-5.68) | -1.59 (-2.31;-0.87) | 0.966 |
|  | Yes | 2831 | 96 (96;97) | 120 | 92 (89;94) | -4.92 (-7.81;-2.03) | -1.53 (-4.35;1.28) |  |
| **By socioeconomic disadvantage** | |  |  |  |  |  |  |  |
| Poverty | No | 96625 | 102 (102;102) | 870 | 98 (97;99) | -3.82 (-4.86;-2.77) | -1.35 (-2.34;-0.37) | 0.767 |
|  | Yes | 26142 | 95 (94;95) | 1101 | 90 (90;91) | -4.19 (-5.19;-3.19) | -1.15 (-2.12;-0.18) |  |
| Parental long-term unemployment* | No | 50432 | 102 (102;102) | 269 | 100 (98;101) | -2.33 (-4.08;-0.57) | -0.32 (-2.01;1.37) | 0.226 |
|  | Yes | 23207 | 96 (96;96) | 882 | 91 (90;92) | -4.79 (-5.88;-3.70) | -1.54 (-2.58;-0.49) |  |
| **By family instability** | |  |  |  |  |  |  |  |
| Parental non-cohabitation | No | 95376 | 101 (101;101) | 906 | 95 (94;96) | -6.02 (-7.15;-4.90) | -1.71 (-2.72;-0.70) | 0.298 |
|  | Yes | 28409 | 97 (97;97) | 1100 | 92 (91;93) | -4.28 (-5.25;-3.31) | -0.98 (-1.93;-0.03) |  |
| ≥3 inter-municipal migrations | No | 120546 | 100 (100;100) | 1893 | 94 (93;94) | -6.53 (-7.29;-5.77) | -1.36 (-2.08;-0.64) | 0.706 |
|  | Yes | 3239 | 101 (100;101) | 113 | 94 (92;97) | -6.05 (-9.05;-3.06) | -0.79 (-3.66;2.07) |  |
| Placement | No | 123279 | 100 (100;100) | 1892 | 94 (93;95) | -6.40 (-7.16;-5.64) | -1.40 (-2.12;-0.68) | **0.021** |
|  | yes | 506 | 90 (88;91) | 114 | 91 (89;94) | 1.64 (-1.58;4.85) | 2.39 (-0.75;5.53) |  |
| **By number of other childhood adversities** | 0 | 61655 | 103 (103;103) | 276 | 101 (99;102) | -2.04 (-3.72;-0.37) | -1.14 (-2.70;0.42) | 0.779 |
|  | 1 | 33274 | 100 (100;100) | 386 | 98 (96;99) | -2.55 (-4.21;-0.90) | -0.80 (-2.36;0.75) |  |
|  | 2 | 17088 | 96 (95;96) | 532 | 92 (91;93) | -3.69 (-5.10;-2.29) | -1.65 (-2.98;-0.32) |  |
|  | 3 | 9673 | 93 (93;94) | 586 | 91 (89;92) | -2.66 (-4.04;-1.28) | -1.06 (-2.41;0.29) |  |
|  | ≥4 | 2095 | 91 (91;92) | 226 | 90 (88;92) | -0.91 (-3.10;1.28) | -0.02 (-2.19;2.15) |  |

Abbreviations: Adj.=adjusted, 95% CI=95% confidence interval.

*The analysis of long-term unemployment was restricted to individuals born between 1998 and 2001.

Estimates are presented as mean differences derived from linear regression analyses. Covariates in the adjusted model for the overall association were birth year, firstborn status, parental highest educational level, maternal age, paternal age, family income, parental citizenship, sibling death, parental somatic illness, sibling somatic illness, sibling psychiatric illness, and parental non-cohabitation. Appendix S1 describes the covariates included in models assessing associations within stratum of childhood adversities. Additionally, the analyses were adjusted for other parental mental disorders than schizophrenia. To assess effect modification, the models included an interaction term between parental mental disorders and the selected childhood adversity and the variables main effects. The p-value is obtained using the Wald test.
